# Pregnancy Complications, Correlation With Placental Pathology and Neonatal Outcomes

**DOI:** 10.3389/fcdhc.2021.807192

**Published:** 2022-03-08

**Authors:** Maria Teresa Loverro, Edoardo Di Naro, Vittorio Nicolardi, Leonardo Resta, Salvatore Andrea Mastrolia, Federico Schettini, Manuela Capozza, Matteo Loverro, Giuseppe Loverro, Nicola Laforgia

**Affiliations:** ^1^ Department of Biomedical Science and Human Oncology, Section of Neonatology and Neonatal Intensive Care, Azienda Ospedaliero-Universitaria Policlinico di Bari, School of Medicine, University of Bari “Aldo Moro”, Bari, Italy; ^2^ Department Interdisciplinary Medicine, Unit of Obstetrics and Gynecology, Azienda Ospedaliero-Universitaria Policlinico di Bari, School of Medicine, University of Bari “Aldo Moro”, Bari, Italy; ^3^ Department of Economics and Finance University of Bari “Aldo Moro”, Bari, Italy; ^4^ Department Emergency and Organ Transplantation, Institute of Pathology, Azienda Ospedaliero-Universitaria Policlinico di Bari, School of Medicine, University of Bari “Aldo Moro”, Bari, Italy; ^5^ Department of Obstetrics and Gynecology, Ospedale dei Bambini “Vittore Buzzi”, University of Milan, Milan, Italy; ^6^ Department of Women and Child Health, Fondazione Policlinico Universitario Agostino Gemelli Istituto di Ricovero e Cura a Carattere Scientifico (IRCCS), Rome, Italy

**Keywords:** great obstetrical syndromes, high-risk pregnancy, low-risk pregnancy, placenta, neonatal outcome

## Abstract

**Purpose:**

We aimed to clarify and contribute to a better comprehension of associations and correlations between placental histological findings, pregnancy evolution, and neonatal outcomes.

**Study Design:**

This is a longitudinal and prospective observational study, performed between May 2015 and May 2019, on 506 pregnant women. Clinical data related to pregnancy outcome, neonatal health status, and placental histology were primarily collected. Twin pregnancies or malformed newborns were excluded and therefore the study was conducted on 439 cases. These cases have been then subdivided into the following study groups: (a) 282 placentas from pathological pregnancies; and, (b) a control group of 157 pregnancies over 33 weeks of gestational age, defined as physiological or normal pregnancies due to the absence of maternal, fetal, and early neonatal pathologies, most of which had undergone elective cesarean section for maternal or fetal indication.

**Results:**

A normal placenta was present in 57.5% of normal pregnancies and in 42.5% of pathological pregnancies. In contrast, placental pathology was present in 26.2% of normal pregnancies and 73.8% of pathological pregnancies. Comparison of the neonatal health status with the pregnancy outcome showed that, among the 191 newborns classified as normal, 98 (51.3%) were born from a normal pregnancy, while 93 (48.7%) were born from mothers with a pathological pregnancy. Among the 248 pathological infants, 59 (23.8%) were born from a mother with a normal pregnancy, while 189 (76.2%) were born from pregnancies defined as pathological.

**Conclusion:**

Placental histology must be better understood in the context of natural history of disease. Retrospective awareness of placental damage is useful in prevention in successive pregnancy, but their early identification in the evolving pregnancy could help in association with biological markers or more sophisticated instruments for early diagnosis.

## Introduction

The placenta is a vital structure for fetal development providing the feto-maternal exchanges of nutrients and catabolic products, and it is strictly influenced by the maternal biohumoral environment which might affect in turn, fetal development.

Although research is already attempting to replace the placenta with an artificial organ, we do not know yet the exact functional framework of this complex natural machine that helps women to generate new lives.

Since it is still difficult to explore the human placenta working principles with the current methods of investigation, histological examination, albeit *a posteriori*, is the only way to understand the complex functions that nature has entrusted to this organ, which functions as lung, kidney, and liver of the fetus, as well as a regulator and stabilizer of fetal biohumoral and endocrine exchange.

Up to the present day, the study of placental histology is the sole instrument enlightening many aspects of placental physiopathology. Nevertheless, it still presents unresolved problems, such as the widespread feedback of classified pathological aspects also in normal pregnancies.

Despite the development and large acceptance of a standardized, reproducible classification system based on previously defined features (1), many details of placental structure and function remain to be elucidated, especially with regard to the influence of maternal conditions and modulation of fetal-neonatal health.

Therefore, pending the development of a machine allowing human embryos to develop outside the womb, it is necessary to understand more about how it works and how we can improve the functioning of the wonderful tool that is the human placenta.

The aim of this study is to investigate the associations between histological examination of the placenta, classified according to the Amsterdam criteria, and the main clinical aspects, both maternal and neonatal, in order to understand to what extent this histological examination reflects placental function and to what extent it can be used for the prognosis and prevention of subsequent pregnancies.

Since the literature has always shown that there is no exact overlap of placental pathological pictures with maternal-fetal-neonatal clinical contexts, stirring up the abovementioned skepticism for the adoption of placental histological findings in clinical practice, we aimed to clarify and contribute to a better comprehension of associations and correlations between placental histological findings, pregnancy evolution, and neonatal outcomes.

## Material and Methods

This is a longitudinal and prospective observational study, performed at the University of Bari “Aldo Moro” between May 2015 and May 2019, on 506 pregnant women enrolled at delivery time after a previous informed consent and ethical committee approval. Clinical data related to pregnancy outcome, neonatal health status, and placental histology were primarily collected. The mode of delivery was mainly cesarean section in 78% (347 cesarean sections and 92 vaginal deliveries) of cases. Twin pregnancies or malformed newborns (67 placentas) were excluded and therefore the study was conducted on 439 cases.

These cases have been then subdivided into the following study groups: (a) 282 placentas from pathological pregnancies; and, (b) a control group of 157 pregnancies over 33 weeks of gestational age, defined as physiological or normal pregnancies due to the absence of maternal, fetal, and early neonatal pathologies, most of which had undergone elective caesarean section for maternal or fetal indication. All these placentas were processed and analyzed by a single operator (RL), who was blinded to maternal and neonatal outcomes and classified them according to the Amsterdam criteria. In addition, placental, cord, and membrane sampling was performed according to the Amsterdam Placental Workshop Group Consensus Statement on placental sampling.

Pregnancies and the respective newborns were classified as normal or pathological based on the presence of one of the criteria listed in **Table 2** for the mother and in **Table 3** for the newborn.

Placental histology has also been classified as normal or pathological, in accordance with the Amsterdam classification, as shown in **Table 4**.

For each type of pathology, the incidence and respective statistical significance of the association with placental pathology has been calculated.

### Statistical Analysis

Statistical analysis was performed using “association analysis” in order to check whether the distribution of the attributes of a certain variable could be defined as dependent or independent from the one of another variable. This analysis was conducted using Pearson’s *χ* ^2^ test and the contingency coefficient and Cramer’s V, which offer a standardized measure of the association level between the variables. Moreover, in order to verify which of the modalities of matched and compared variables has determined the strongest degree of association between them, the cell *χ* ^2^ tests have been calculated for each cell of intersection of the contingency tables used.

### Definition of Variables

Two subgroups of pregnancies have been identified: physiological pregnancies and pathological pregnancies, characterized by abnormal conditions that could impair the health or the life of the mother and fetus, such as maternal diabetes, oligohydramnios (AFI <5), clinically significant uterine blood loss, central placenta previa, preeclampsia, stained amniotic fluid, intrauterine fetal death, hypertension, intrauterine growth retardation, and premature rupture of membranes.

In turn, also newborns were divided into normal and pathological, according to the presence or absence of neonatal weight lower than 1,500 g, neonatal sepsis, respiratory distress, neonatal resuscitation, small gestational age (SGA), neonatal hospitalization longer than 10 days, preterm infant, and neonatal deaths. A SGA fetus was defined as a fetus with an estimated fetal weight or an abdominal circumference below the 10th percentile of given reference ranges.

The placentas have also been divided into normal and pathological. Each pathological placenta was grouped by a single pathologist (RL) into the main groups, according to the Amsterdam classification (1) and corresponding to: infection, maternal malperfusion, fetal malperfusion, chorangiosis, intervillous hemorrhage, and other.

The histological results of the placenta corresponding to maternal or neonatal data were then associated with each corresponding pregnancy.

## Results

Among the 439 pregnancies considered eligible for analysis, 157 (35.8%) were physiological pregnancies randomly enrolled in 4 years and 282 (64.2%) were pathological pregnancies, with a mean annual enrolment of 72 physiological pregnancies treated in the Department of Obstetrics and Gynecology (**Table 1**).

**Table 1:** Distribution of placental pathologies and neonatal outcomes by type of pregnancy.

Placenta	Pregnancy			Newborns	Pregnancy		
	Normal	Pathological	Total		Normal	Pathological	Total
**Normal**	77 (57.5%) (49%)	57 (42.5%) (20.2%)	134 (100%) (30.5%)	**Normal**	98 (51.3%) (62.4%)	93 (48.7%) (33%)	191 (100%) (43.5%)
**Pathological**	80 (26.2%) (51%)	225 (73.8%) (79.8%)	305 (100%) (69.5%)	**Pathological**	59 (23.8%) (37.6%)	189 (76.2%) (67%)	248 (100%) (56.5%)
**Total**	157 (35.8%) (100%)	282 (64.2%) (100%)	439 (100%) (100%)	**Total**	157 (35.8%) (100%)	282 (64.2%) (100%)	439 (100%)

A normal placenta was present in 57.5% of normal pregnancies and in 42.5% of pathological pregnancies.

In contrast, placental pathology was present in 26.2% of normal pregnancies and 73.8% of pathological pregnancies.

Comparison of the neonatal health status with the pregnancy outcome showed that, among the 191 newborns classified as normal, 98 (51.3%) were born from a normal pregnancy, while 93 (48.7%) were born from mothers with a pathological pregnancy.

Among the 248 pathological infants, 59 (23.8%) were born from a mother with a normal pregnancy, while 189 (76.2%) were born from pregnancies defined as pathological (**Table 1**).

The incidence of the various pregnancy diseases detected in the 282 pathological pregnancies is shown in **Table 2**. The most frequent pregnancy pathology was the premature rupture of membranes before 37 weeks (72 cases, 25.5%), followed by 55 cases (19.5%) of IUGR and then 44 cases of hypertension (15.6%), 27 cases (9.6%) of oligohydramnios (AFI <5), in the absence of premature rupture of membranes), 23 cases of gestational diabetes (8,2%), 15 cases of central placenta previa (5.3%), 13 cases of preeclampsia (4.6%), 13 cases of meconium stained amniotic fluid grade II or III (4.6%), 12 cases of profuse blood loss due to partial or total placental abruption (4.3%), and, finally, 8 cases (2.8%) of intrauterine fetal death.

**Table 2:** Distribution of pathological pregnancy outcomes and related pathologies.

Pregnancy complications	Number (%)
Maternal diabetes	23 (8.2%)
Oligohydramnios (AFI <5)	27 (9.6%)
Uterine blood loss from placental abruption	12 (4.3%)
Placenta previa/accreta	15 (5.3%)
Preeclampsia	13 (4.6%)
Stained amniotic fluid	13 (4.6%)
Stillbirth	8 (2.8%)
Maternal hypertension	44 (15.6%)
Fetal intrauterine growth retardation IUGR	55 (19.5%)
P-PROM premature rupture of membrane (<37 weeks)	72 (25.5%)
**Total pathological pregnancies**	**282 (100%)**

Among neonatal complications (**Table 3**), the most frequent has been the birth of 68 premature neonates (27.4%), 64 small gestational age (SGA) infants (25.8%), followed by 36 cases (14.5%) of infants in need of primary resuscitation, 29 cases (11.7%) of respiratory distress, 23 cases of sepsis (9.3%), 19 cases (7.7%) of neonatal hospitalisation longer than 10 days, and 9 (3.6%) cases of neonatal death.

**Table 3:** Distribution of neonatal complications.

Neonatal complications	Number (%)
Premature neonates	68 (27.4%)
Neonatal sepsis	23 (9.3%)
Neonatal respiratory distress	29 (11.7%)
Neonatal resuscitation	36 (14.5%)
Small for gestational age neonate	64 (25.8%)
Neonatal hospitalization longer than 10 days	19 (7.7%)
Neonatal death	9 (3.6%)
**Total pathological births**	248 (100%)

With regard to placental histology, 134 pregnancies (30.5%) had a normal placenta, while 305 (69.5%) had placentas defined as pathological due to histological lesions (**Table 1**), among them 80 (26.2%) were from physiological pregnancies and 225 (79.8%) from pathological pregnancies.

Among placental lesions (**Table 4**), the most frequent observed condition is maternal malperfusion found in 144 patients (47.2%), followed by infections diagnosed in 71 patients (23.3%), chorangiosis and intervillous hemorrhage, both identified in 29 patients (9.5%), fetal malperfusion in 8 cases (2.6%), and, finally, different types of pathologies, defined here as “other” in 24 placentas (7.9%).

**Table 4:** Distribution of type of placental pathology.

Types of placental histologic anomaly	Number (%)
Infection	71 (23.3%)
Maternal malperfusion	144 (47.2%)
Fetal malperfusion	8 (2.6%)
Chorangiosis	29 (9.5%)
Intervillous hemorrhage	29 (9.5%)
Others	24 (7.9%)
**Total pathological placentas**	**305 (100%)**

### Analysis of Association of Placental Histology, Pregnancy, and Neonatal Outcomes

In order to understand whether the results of histological examination of the placenta were significantly associated with pregnancy or neonatal outcomes, an association analysis was conducted between the type of pregnancy (physiological or pathological), placental histology (normal or pathological) and early neonatal health conditions (normal or pathological).

Preliminarily, the analysis of association between pregnancy and neonatal outcomes has been performed, displaying a good level of association between the type of pregnancy and neonatal outcomes, as confirmed by both the *χ* ^2^ tests (*p* < 0.0001) and the contingency coefficient and Cramer’s V, confirming at the same time a logical assumption that a physiological pregnancy is associated with a disease-free placenta.

Subsequently, the analysis of the degree of association between the pregnancy outcome and the neonatal diseases considered here showed that normal infants displayed a significant degree of association with normal pregnancies (*χ* ^2^ = 12.91, *p*-value = 0.0003) and that, among all neonatal diseases, infants with SGA reported a significant degree of association with pathologic pregnancies (*χ* ^2^ = 4.69, *p*-value = 0.0303).

Indeed, SGA has a significantly higher incidence in pathological pregnancies (19.5%) than in physiological pregnancies (5.7%), while healthy neonates have a significantly higher incidence in normal pregnancies (62.4%) compared with pathological pregnancies (33%).

The other neonatal pathologies, however, do not present statistically significant differences between pathological and normal pregnancies.

To be precise, premature neonates are present in 12.7% of physiological pregnancies and 17% of pathological pregnancies, while neonatal sepsis shows an incidence of 3.8% in normal pregnancies and 6% in pathological pregnancies. In addition, the incidence of neonatal respiratory distress resulted higher in pathological pregnancies (7.8%) than in physiological pregnancies (4.5%), although the difference is not significant.

Finally, the incidence of neonatal resuscitation is substantially comparable between normal and pathological pregnancies (8.3% and 8.2%, respectively), while neonatal hospitalization longer than 10 days showed a higher incidence in pathological pregnancies (5.7%) than in physiological ones (1.9%), without a significant difference probably due to small number of neonates experiencing a long hospital stay after a normal pregnancy.

Likewise, intrauterine deaths reported a relatively higher incidence in pathological pregnancies (2.8%) than in normal pregnancies (only 1 case, 0.6%) (**Table 5**).

**Table 5:** Neonatal conditions according to pregnancy conditions.

Neonatal conditions	Normal pregnancy	Pathological pregnancy
Premature neonates	20 (12.7%)	48 (17%)
Sepsis	6 (3.8%)	17 (6%)
Respiratory distress at birth	7 (4.5%)	22 (7.8%)
Neonatal intensive care	13 (8.3%)	23 (8.2%)
SGA Small gestational age	9 (5.7%)	55 (19.5%)
Neonatal hospitalization >10 days	3 (1.9%)	16 (5.7%)
Neonatal death	1 (0.6%)	8 (2.8%)
Normal neonates	98 (62.4%)	93 (33%)
**Total**	**157 (100%)**	**282 (100%)**

### Analysis of Association Between Neonatal Outcomes and Maternal Diseases

The analysis of the association between neonatal outcomes and maternal pathologies appeared significant, as shown both by the *χ* ^2^ tests (*p* < 0.0001) and by the contingency coefficient and Cramer’s V.

Therefore, the distribution and significance of the association between the different components of the two variables under consideration was specifically analyzed (**Table 6**), reflecting a significant association between specific neonatal outcomes and maternal pathologies, both displaying significantly higher joint frequencies than simple action due to chance.

**Table 6:** Contingency table between neonatal outcomes and maternal diseases.

Neonatal outcome	Pregnancy complications											
	Maternal diabetes	Oligohydramnios	Uterine bleeding	Placenta previa	Pre-eclampsia	Meconium stained amniotic fluid	Intrauterine fetal death	Maternal hypertension	IUGR	Normal pregnancies	PROM	Total
**Premature neonates**	–	6 (8.82%) (22.22%)	4 (5.88%) (33.33%)	4 (5.88%) (26.67%)	6 (8.82%) (46.15%)	1 (1.47%) (7.69%)	–	9 (13.24%) (20.45%)	5 (7.35%) (9.09%)	20 (29.41%) (12.74%)	13 (19.12%) (18.06%)	**68 (15.5%)**
**Sepsis**	1 (4.4%) (4.4%)	2 (8.7%) (7.4%)	–	–	1 (4.4%) (7.7%)	2 (8.7%) (15.4%)	–	3 (13%) (6.8%)	–	6 (26.1%) (3.8%)	8 (34.8%) (11.1%)	**23 (5.2%)**
**Neonatal respiratory distress**	3 (10.3%) (13%)	1 (3.5%) (3.7%)	–	3 (10.3%) (20%)	3 (10.3%) (23.1%)	–	–	3 (10.3%) (6.8%)	3 (10.3%) (5.5%)	7 (24.1%) (4.5%)	6 (20.7%) (8.3%)	**29 (6.6%)**
**Neonatal intensive care**	1 (2.8%) (4.4%)	2 (5.6%) (7.4%)	–	2 (5.6%) (13.3%)	1 (2.8%) (7.7%)	1 (2.8%) (7.7%)	–	4 (11.1%) (9.1%)	6 (16.7%) (10.9%)	13 (36.1%) (8.3%)	6 (16.7%) (8.3%)	**36 (8.2%)**
**SGA**	3 (4.7%) (13%)	5 (7.8%) (18.5%)	2 (3.1%) (16.7%)	1 (1.6%) (6.7%)	1 (1.6%) (7.7%)	–	–	7 (10.9%) (15.9%)	25 (39.1%) (45.5%)	9 (14.1%) (5.7%)	11 (17.2%) (15.3%)	**64 (14.6%)**
**Neonatal hospitalization >10 days**	–	1 (5.3%) (3.7%)	–	–	1 (5.3%) (7.7%)	2 (10.5%) (15.4%)	–	4 (21.1%) (9.1%)	6 (31.6%) (10.9%)	3 (15.8%) (1.9%)	2 (10.5%) (2.8%)	**19 (4.3%)**
**Neonatal death**	–	–	–	–	–	–	8 (88.9%) (100%)	–	–	1 (11.1%) (0.6%)	–	**9 (2.1%)**
**Normal**	15 (7.9%) (65.2%)	10 (5.2%) (37%)	6 (3.1%) (50%)	5 (2.6%) (33.3%)	–	7 (3.7%) (53.9%)	–	14 (7.3%) (31.8%)	10 (5.2%) (18.2%)	98 (51.3%) (62.4%)	26 (13.6%) (36.1%)	**191 (43.5%)**
**Total**	**23 (5.2%) (100%)**	**27 (6.2%) (100%)**	**12 (2.7%) (100%)**	**15 (3.4%) (100%)**	**13 (3%) (100%)**	**13 (3%) (100%)**	**8 (1.8%) (100%)**	**44 (10%) (100%)**	**55 (12.5%) (100%)**	**157 (35.7%) (100%)**	**72 (16.4%) (100%)**	**439 (100%) (100%)**

Specifically, a high association was identified between the intrauterine diagnosis of small gestational age and the neonatal diagnosis of intrauterine growth retardation (IUGR) (*χ* ^2^ = 34.69, *p*-value <0.0001), confirming the well-known predictive value of prenatal biometric ultrasound screening. Significant has proven to be the association between normal pregnancies and normal neonates (*χ* ^2^ = 11.61, *p*-value = 0.0007), between premature neonates and preeclampsia (*χ* ^2^ = 7.60, *p*-value = 0.0058), between neonatal hospital stay longer than 10 days and IUGR intrauterine growth retardation (*χ* ^2^ = 5. 27, *p*-value = 0.0217), respiratory neonatal distress and preeclampsia (*χ* ^2^ = 5.16, *p*-value = 0.0231), neonatal sepsis and PROM premature rupture of membrane (*χ* ^2^ = 4.5, *p*-value = 0.0339), and, finally, respiratory distress at birth and placenta previa (*χ* ^2^ = 3.93, *p*-value = 0.0475).

The correlation of other possible correlated variants in the present study did not reveal any significant degree of association.

### Analysis of Association Between Placental Pathology and Pregnancy Outcomes

Therefore, considering that, overall, the incidence of neonatal pathologies is correlated to the presence or absence of pregnancy pathology, we wondered if and to what extent this association is influenced by or could be related with a normal or pathological placenta. For this reason, we have analyzed the association between the placental histology, classified according to the Amsterdam criteria, the pregnancy outcome and the neonatal health status.

This analysis has demonstrated the presence of a significant association between placental pathology and the pregnancy outcome, as confirmed by the *χ* ^2^ test (*p* < 0.0001) and the Contingency Coefficient and Cramer’s V.

Therefore, the detailed analysis of the distribution of the placental pathologies related to the course of pregnancy (**Table 7**), has highlighted a high degree of association between normal placentas and physiological pregnancies (*χ* ^2^ = 17. 64, *p*-value < 0.0001), between placental maternal malperfusion and pathological pregnancies (*χ* ^2^ = 8.78, *p*-value = 0.0030) and between intervillous hemorrhage and physiological pregnancies (*χ* ^2^ = 4.24, *p*-value = 0.0396).

**Table 7:** Distribution of placental pathologies in relation to pregnancy outcome.

Placental pathologies	Normal pregnancies	Pathological pregnancies
Infection	25 (15.9%)	46 (16.3%)
Maternal malperfusion	23 (14.7%)	121 (42.9%)
Fetal malperfusion	2 (1.3%)	6 (2.1%)
Chorangiosis	5 (3.2%)	24 (8.5%)
Intervillous hemorrhage	17 (10.8%)	12 (4.3%)
Other	8 (5.1%)	16 (5.7%)
Normal placenta	77 (49%)	57 (20.2%)
**Total**	**157 (100%)**	**282 (100%)**

Overall, the absence of placental abnormalities was found in 49% of normal pregnancies, compared with 20.2% of pathological pregnancies.

Maternal malperfusion is present in 14.7% of physiological pregnancies and 42.9% of pathological pregnancies, making it the most important placental lesion with a possible active role in determining the pregnancy outcome. Its presence in a non-negligible percentage in physiological pregnancies deserves careful consideration and calls, perhaps, for a better pathological significance.

Finally, intervillous hemorrhage is present in 10.8% of normal pregnancies and 4.3% of pathological pregnancies. This difference leads increasingly more to considering intervillous hemorrhage as a paraphysiological phenomenon of normal pregnancy arrangement, whose intensity is not such as to result in fetal hypoxia.

As far as other placental pathologies are concerned, they do not show significant differences between normal and pathological pregnancies. Among these we note that chorangiosis is present in 3.2% of normal pregnancies and in 8.5% of pathological pregnancies, infection in 15.9% of physiological pregnancies and in 16.3% of pathological pregnancies, fetal malperfusion in 1.3% of physiological pregnancies and in 2.1% of pathological pregnancies. Finally, placental pathologies defined as “other” have a 5.1% incidence in physiological pregnancies and 5.7% in pathological pregnancies (**Table 7**).

### Analysis of Association Between Placental and Maternal Pathologies

In order to further investigate the interrelations between the factors examined here, it appeared important to conduct an association analysis between placental pathologies and pregnancy pathologies.

The degree of association between the two variables was significant, as shown both by the *χ* ^2^ tests (*p* < 0.0001) and by the contingency coefficient and Cramer’s V.

The analysis of the distribution of the different modalities of the two variables analyzed (**Table 8**), displayed significant associations between certain types of placental disorders and maternal diseases, corresponding to significantly higher joint frequencies than the simple action by chance.

**Table 8:** Contingency table between placental and maternal pathologies.

Placental disorders	Normal pregnancy	Pregnancies complicated by maternal disease											
		Maternal diabetes	Oligoidramnios	Uterine bleeding	Placenta previa	Preeclampsia	Meconium-stained amniotic fluid	Intrauterine fetal death	Maternal hypertension	IUGR	Normal pregnancy	P-PROM	Total
Infection	25 (35.2%) (15.9%)	–	6 (8.5%) (22.2%)	2 (2.8%) (16.7%)	3 (4.2%) (20.0%)	–	5 (7.0%) (38.5%)	3 (4.2%) (37.5%)	3 (4.2%) (6.8%)	2 (2.8%) (3.6%)	25 (35.2%) (15.9%)	22 (31.0%) (30.6%)	71 (16.2%)
Maternal malperfusion	23 (16.0%) (14.7%)	5 (3.5%) (21.7%)	7 (4.9%) (25.9%)	4 (2.8%) (33.3%)	2 (1.4%) (13.3%)	11 (7.6%) (84.6%)	2 (1.4%) (15.4%)	2 (1.4%) (25.0%)	34 (23.6%) (77.3%)	34 (23.6%) (61.8%)	23 (16.0%) (14.7%)	20 (13.9%) (27.8%)	144 (32.8%)
Fetal malperfusion	2 (25.0%) (1.3%)	–	2 (25.0%) (7.4%)	–	–	–	–	–	–	4 (50.0%) (7.3%)	2 (25.0%) (1.3%)	–	8 (1.8%)
Chorangiosis	5 (17.2%) (3.2%)	13 (44.8%) (56.5%)	2 (6.9%) (7.4%)	–	–	–	1 (3.5%) (7.7%)	–	2 (6.9%) (4.6%)	4 (13.8%) (7.3%)	5 (17.2%) (3.2%)	2 (6.9%) (2.8%)	29 (6.6%)
Intervillous hemorrhage	17 (58.6%) (10.8%)	2 (6.9%) (8.7%)	1 (3.5%) (3.7%)	–	2 (6.9%) (13.3%)	1 (3.5%) (7.7%)	1 (3.5%) (7.7%)	–	2 (6.9%) (4.6%)	–	17 (58.6%) (10.8%)	3 (10.3%) (4.2%)	29 (6.6%)
Other	8 (33.3%) (5.1%)	–	3 (12.5%) (11.1%)	1 (4.2%) (8.3%)	2 (8.3%) (13.3%)	1 (4.2%) (7.7%)	1 (4.2%) (7.7%)	2 (8.3%) (25.0%)	–	1 (4.2%) (1.8%)	8 (33.3%) (5.1%)	5 (20.8%) (6.9%)	24 (5.5%)
Normal placenta	77 (57.5%) (49.0%)	3 (2.2%) (13.0%)	6 (4.5%) (22.2%)	5 (3.7%) (41.7%)	6 (4.5%) (40.0%)	–	3 (2.2%) (23.1%)	1 (0.8%) (12.5%)	3 (2.2%) (6.8%)	10 (7.5%) (18.2%)	77 (57.5%) (49.0%)	20 (14.9%) (27.8%)	134 (30.5%)
**Total**	157 (35.8%) (100%)	23 (5.24%) (100%)	27 (6.2%) (100%)	12 (2.7%) (100%)	15 (3.4%) (100%)	13 (3.0%) (100%)	13 (3.0%) (100%)	8 (1.8%) (100%)	44 (10.0%) (100%)	55 (12.5%) (100%)	157 (35.8%) (100%)	72 (16.4%) (100%)	439 (100%) (100%)

In particular, a high association was found between chorioangiosis and maternal diabetes (*χ* ^2^ = 86.75, *p*-value < 0.0001), maternal malperfusion and maternal hypertension (*χ* ^2^ = 26.53, *p*-value < 0.0001), normal placenta and normal pregnancies (*χ* ^2^ = 17. 64, *p*-value < 0.0001), maternal malperfusion and IUGR (*χ* ^2^ = 14.12, *p*-value = 0.0002), maternal malperfusion and preeclampsia (*χ* ^2^ = 10.64, *p*-value = 0.0011), infection and PROM (*χ* ^2^ = 9.21, *p*-value = 0. 0024), fetal malperfusion and IUGR (*χ* ^2^ = 8.97, *p*-value = 0.0028), other placental diseases and fetal demise (*χ* ^2^ = 5.58, *p*-value = 0.0181), fetal malperfusion and oligohydramnios (*χ* ^2^ = 4. 62, *p*-value = 0.0316), intervillous hemorrhage and normal pregnancies (*χ* ^2^ = 4.24, *p*-value = 0.0396), and infection and amniotic fluid meconium stained (*χ* ^2^ = 3.99, *p*-value = 0.0457).

The other possible associations did not show a significant degree of association.

### Analysis of Association Between Neonatal Outcomes and Placental Disease

The analysis of the association between neonatal outcomes and placental disease showed a significant degree results, as shown by both *χ* ^2^ tests (*p* < 0.0001), contingency coefficient and Cramer’s V.

A specific analysis of the distribution of placental pathologies in relation to neonatal pathologies (**Table 9**) reveals that some of them are significantly higher than a simple action of chance.

**Table 9:** Contingency table between neonatal outcomes and placental pathologies.

Neonatal outcomes	Placental pathologies							
	Infection	Maternal malperfusion	Fetal malperfusion	Chorangiosis	Intervillous hemorrhage	Other	Normal placenta	Total
**Premature neonates**	15 (22.1%) (21.2%)	25 (36.8%) (17.3%)	1 (1.5%) (12.5%)	2 (2.9%) (6.9%)	9 (13.2%) (31.3%)	2 (2.9%) (8.3%)	14 (20.6%) (10.5%)	**68 (100%) (15.5%)**
**Sepsis**	15 (65.2%) (21.1%)	5 (21.7%) (3.5%)	–	1 (4.3%) (3.5%)	–	2 (8.7%) (8.3%)	–	**23 (100%) (5.2%)**
**Neonatal respiratory distress**	3 (10.3%) (4.2%)	10 (34.5%) (6.9%)	–	4 (13.8%) (13.8%)	2 (6.9%) (6.9%)	3 (10.3%) (12.5%)	7 (24.1%) (5.2%)	**29 (100%) (6.6%)**
**Neonatal intensive care**	6 (16.7%) (8.5%)	12 (33.3%) (8.3%)	–	1 (2.8%) (3.5%)	1 (2.8%) (3.5%)	3 (8.3%) (12.5%)	13 (36.1%) (9.7%)	**36 (100%) (8.2%)**
**SGA small gestational age**	6 (9.4%) (8.5%)	33 (51.6%) (22.9%)	4 (6.3%) (50.0%)	5 (7.8%) (17.2%)	3 (4.7%) (10.3%)	1 (1.6%) (4.2%)	12 (18.8%) (9.0%)	**64 (100%) (14.6%)**
**Neonatal hospitalization >10 days**	2 (10.5%) (2.8%)	8 (42.1%) (5.6%)	1 (5.3%) (12.5%)	1 (5.3%) (3.5%)	–	1 (5.3%) (4.2%)	6 (31.6%) (4.5%)	**19 (100%) (4.3%)**
**Neonatal death**	3 (33.3%) (4.2%)	3 (33.3%) (2.1%)	–	–	–	2 (22.2%) (8.3%)	1 (11.1%) (0.8%)	**9 (100%) (2.1%)**
**Normal**	21 (11.0%) (29.6%)	48 (25.1%) (33.3%)	2 (1.0%) (25.0%)	15 (7.9%) (51.7%)	14 (7.3%) (48.3%)	10 (5.2%) (41.7%)	81 (42.4%) (60.5%)	**191 (100%) (43.5%)**
**Total**	**71 (16.2%) (100%)**	**144 (32.8%) (100%)**	**8 (1.8%) (100%)**	**29 (6.6%) (100%)**	**29 (6.6%) (100%)**	**24 (5.5%) (100%)**	**134 (30.5%) (100%)**	**439 (100%) (100%)**

In particular, there is a high association between neonatal sepsis and placental infections (*χ* ^2^ = 34.21, *p*-value < 0.0001), together with a similar significant association between normal neonates and normal placentas (*χ* ^2^ = 8.84, *p*-value = 0. 0030), small gestational age and fetal malperfusion (*χ* ^2^ = 6.89, *p*-value = 0.0087), small gestational age and maternal malperfusion (*χ* ^2^ = 6.87, *p*-value = 0.0088), neonatal deaths and the grouping of other placental diseases named “other” (*χ* ^2^ = 4.62, *p*-value = 0.0316), and between premature neonates and intervillous hemorrhage (*χ* ^2^ = 7.60, *p*-value = 0.0058).

For the remaining matched pairs of modalities, there was no significant degree of association between neonatal outcomes and the presence of placental pathologies.

## Discussion

The role of human placenta continues to be the subject of interpretative debate, despite the fact that ever greater progress is being made in the development of an artificial placenta (2).

For this reason, the histological study of the placenta continues to have poor implication in clinical practice and a difficult place in forensic medicine.

Human placenta is a key organ in “programming the fetus for later disease” (3), and, as a consequence, it might be even more important in programming newborn disease.

The primary and useful approach in placental clinical application is the study of its histological lesions. However, despite the large number of articles published and the widespread acceptance of the “Amsterdam Classification,” this precious diagnostic tool has not found a wide application into neonatal and in obstetric care of successive pregnancies yet.

This difficulty may be due to various reasons such as the finding, albeit in varying percentages, of the same lesion in both normal and pathological pregnancies, leading to a nonunique clinical interpretation.

Moreover, although there is an adequate nosology of placental lesions, the multifactorial nature of pregnancy evolution does not allow us to understand to what extent the maternal pathophysiology can affect placental function and, above all, to what extent pathological placenta can influence fetal-neonatal health.

Many pathological maternal and neonatal conditions have been associated with pathological placental histology, although few are based on reliable association analyses.

The instrument adopted here to better understand the relationship between the maternal and placental pathology, as well as those between placental and neonatal diseases, is the analysis of association between variables and, specifically in our case, between maternal, placental, and neonatal pathology (4–8).

The association analysis, adopted in the present study, has provided us with data that may help to better understand the clinical use of histological findings and, at the same time, may shed further light on the significance of these lesions in both physiological and pathological pregnancy.

### Influence of Placental Pathology on Pregnancy Outcome

In pregnancies classified as normal, only 49% did not display any placental alterations, while 15.9% of placentas had infections, 14.7% maternal malperfusion, and 10.3% intervillous hemorrhage, to mention just the most representative.

Although present in a nonnegligible proportion and significantly lower than the respective incidence of pathological pregnancies (73.8%), this percentage of pathological placentas in normal pregnancy could be interpreted as paraphysiological which, for reasons still unknown, does not result in a clinical pathology. Nevertheless, the nonnegligible extent of these lesions poses a problem, addressed in the literature mainly for placental infections, of graduation of the intensity of the lesion, the real functional placental impairment.

In our experience, only intervillous hemorrhage is the histological lesion significantly present (*χ* ^2^ = 4.24, *p*-value = 0.0396) in normal pregnancies. This incidence is significantly higher than the one of pathological pregnancies (4.3%), reaffirming the quasi-physiological significance of this lesion. Presumably, the other lesions that remain nonsignificantly associated are not so frequent or intense enough to induce a pathological outcome of the pregnancy.

In order to better understand the reciprocal influence of pregnancy disorders, placental abnormalities, and neonatal disease, we have analyzed the relative mutual association and the reciprocal influence.

In the grouping of pathological pregnancies, the analysis of the association between the various types of placental and maternal pathologies demonstrated a statistical significance among some of the variables taken into account, due to higher joint frequencies than chance, as shown in **Table 8**.

In the context of maternal pathology, placenta previa and uterine bleeding are characterized by and have in common many placental lesions, such as infection, maternal malperfusion, intervillous hemorrhage, as well as some placental lesions grouped under the name of “others,” although without any significantly characterizing incidence compared with physiological pregnancies.

The small group of cases with intrauterine fetal death is characterized by a significant association only with the group of other placental pathologies (“other”) (*χ* ^2^ = 5.58, *p*-value = 0.0181), underlining the great variability of causes of intrauterine death in the third trimester of pregnancy and, therefore, the need to perform an autopsy in any case to clarify the fairly heterogenous pathogenetic mechanism.

The sonographic diagnosis of oligohydramnios also displayed a significant association with malperfusion (*χ* ^2^ = 4.62, *p*-value = 0.0316), while meconium-stained amniotic fluid (*χ* ^2^ = 3.99, *p*-value = 0.0457) has been significantly associated with placental infection and intervillous hemorrhage, suggesting in both cases the presence of unquantifiable reduced oxygenation of the fetus by the placenta.

Preeclampsia has been significantly associated to the maternal malperfusion (*χ* ^2^ = 10.64, *p*-value = 0.0011) as a characterizing placental lesion. The key role of the altered placental vascularization is also confirmed by the significant association between the intrauterine diagnosis of intrauterine growth retardation (IUGR) and maternal malperfusion (*χ* ^2^ = 14.12, *p*-value = 0.0002), underlining the usefulness of a very early diagnosis of impaired placental function.

Association between fetal IUGR and fetal malperfusion appear highly significant (*χ* ^2^ = 8.97, *p*-value = 0.0028) underlying the crucial role of placental disorders in fetal growth defects due to chronic fetal hypoxia. The poor villi development with a consequent hypoplasia and poor branching of the villous tree, the increased syncytial nodes, and formation of vasculo-syncytial membranes, are indeed the true architectural elements, distinctive of the vascular-based fetal growth pathologies, characterizing the fetal malperfusion.

It would therefore be desirable to identify this condition at an early stage, in order to implement all the therapeutic measures that research is trying to validate.

The significant association between the reduction of amniotic fluid and the placental fetal vascular malperfusion (*χ* ^2^ = 4.62, *p*-value = 0.0316), emphasizes a reduced or absent perfusion of the villous parenchyma, characterized by vascular stasis, ischemia, and in some cases, thrombosis, associated to reduced placental function.

A striking high-degree association, particularly significant, has been the one between gestational diabetes and placental chorangiosis (*χ* ^2^ = 86.75, *p*-value < 0.0001) found in 56.5% of our gestational diabetes cases.

Chorangiosis is characterized by an increase in the number of fetal capillaries in the terminal villi in noninfarcted areas of the placenta.

It represents a compensation mechanism in some cases of chronic hypoxia, which, strangely enough, is present from the early stages of pregnancy in the presence of completely asymptomatic glycemic dysmetabolism. This coincidence is clinically unexpected but seems plausible from a biological point of view.

Indeed, pregnancies complicated by diabetes show an increase in human placental lactogen already at the very early stages of pregnancy with the induction of a “diabetogenic state,” leading to a higher glucose availability for the fetus (9). This is associated to an increased production of insulin-like growth factor and growth factors promoting tissue growth (9), and this mechanism may be the basis for the placental changes observed in chorangiosis.

It would therefore be desirable to diagnose dysmetabolism in a very early stage of pregnancy, also by means of the tools offered by recent omics sciences (10).

The analysis of the distribution of the different modalities of the two variables analyzed, maternal and placental pathologies (**Table 8**) has revealed further significant associations. In fact, the association between premature rupture of membranes and placental infection (*χ* ^2^ = 9.21, *p*-value = 0.0024), corresponds to significantly higher joint frequencies than the simple action by chance.

Premature rupture of membranes may result from placental-fetal inflammation syndrome, maternal placental malperfusion, or both, irrespective of gestational age and of the interval between rupture of membranes and time of delivery (11).

Preterm birth has resulted to be significantly associated with certain Amsterdam classification placental pathologies, manly with acute and chronic inflammation and/or altered early maternal perfusion, often resulting from chorioamnionitis.

According to the literature, the incidence of placental infections, presenting as chorioamnionitis, occurs in 32% of preterm deliveries, compared with 10% of normal pregnancies (12).

As far as the problem of premature birth is concerned, we detected a 23% rate of placental infection, without any difference between normal and pathological pregnancies (**Table 7**). Moreover, our results did not identify any significant association between placental infection and premature birth, which is inconsistent with the literature (13, 14). These differences may be related to the mode of recruitment (pregnancies over 32 weeks) and, in large part, elective cesarean section.

### Vascular Implications in Placental Pathology and Pregnancy Outcomes

Maternal vascular malperfusion has been reported in the literature with a reported incidence varying between 35% (15), 19.7% (14), and 1.5% (16).

In our experience, maternal malperfusion has been present in 47.2% of cases, accounting for a large number of pathological pregnancies, especially IUGR and preeclampsia, enrolled in the present study.

Therefore, inadequate remodeling of the spiral artery or pathology of the spiral arteries (decidual vasculopathy), is the main factor inducing a placental picture of maternal vascular malperfusion, strictly associated with preeclampsia, IUGR (17), with possible development of cerebral palsy or neurocognitive abnormalities at school age, especially in very low birth weight infants (<1 kg) (18).

For this reason and the potential severity of the outcomes mentioned above, there has been in the last decades an effort to prevent or reduce the inadequate remodeling of the spiral arteries primarily by the administration of low-dose aspirin during pregnancy (19–21).

### Correlation of Early Neonatal Disease to Placental Pathology

The present study demonstrates unequivocally that in the absence of placental lesions, not only does pregnancy develop in a normal manner but newborns are also healthy.

As shown by the association analysis, a normal placenta is associated with a high probability to deliver a healthy baby (*χ* ^2^ = 8.84, *p*-value = 0.0030), underlining the active role of the placenta during pregnancy in preserving the fetal well-being.

In contrast, but according to the same approach, neonatal sepsis is strongly and significantly associated with placental infection (*χ* ^2^ = 34.21, *p*-value < 0.0001).

In our experience, no placental lesion has resulted to be significantly associated with an increased incidence of perinatal asphyxia, and this reflects the vagueness of the literature data, according to which perinatal asphyxia has been associated with placental lesions affecting the vascular supply to the fetus, such as umbilical cord complications (interrupted velamentous vessels, cord laceration, hypercoiled cord, cord hematoma), chorioamnionitis with fetal vasculitis, fetal thrombotic vasculopathy, ascending intrauterine infection, and reduced maternal vascular perfusion with low Apgar scores at 1 and 5 min (22, 23).

Therefore, we can reaffirm that the role of placental lesions in perinatal asphyxia with neonatal death exists, but it is not exclusive, considering that the role of chronic hypoxic trauma *in utero* is difficult to be evaluated by histological examination, as it is related to a functional factor represented by the ability of the placenta to compensate the hypoxic conditions.

Maternal malperfusion was significantly associated with a neonatal diagnosis of small gestational age at birth (*χ* ^2^ = 6.87, *p*-value = 0.0088).

Neonatal sepsis is significantly associated with increased incidence of placental inflammation, classified as villitis of unknown origin and chorioamnionitis (24, 25).

In our experience, neonatal sepsis was significantly associated with placental infection, which was present in 65.2% of neonatal sepsis (*χ* ^2^= 34.21, *p*-value < 0.0001), showing a strong dependence between the two conditions.

Similarly, there is a significant association between neonatal deaths and the grouping of placental pathologies called “other” (*χ* ^2^ = 4.62, *p*-value = 0.0316), although placental and neonatal infection were present with an important, but nonsignificant, incidence of 33.3%. Therefore, we believe that the limitation of significance is due to a nonhomogeneous group of placental lesions and to the small number of cases, even though placental pathology has a primary role in the case of neonatal death, clearly in the context of a multifactorial nature of this event.

## Conclusion

In conclusion, placental histology must be better understood in the context of natural history of the disease.

Retrospective awareness of placental damage is useful in prevention of successive pregnancy, but their early identification in evolving pregnancy could be very useful if we have biological markers or more sophisticated instruments for early diagnosis.

As a result, we believe that the efforts of research should be directed towards identification of technological innovations, capable of identifying early placental pathologies. Unfortunately, this will not happen without the help of robust biomarkers, capable of predicting the onset of the placenta disease early in pregnancy. Future research in these areas will be critical for filling this gap and will open a new era of placental therapy.

## Data Availability Statement

The raw data supporting the conclusions of this article will be made available by the authors, without undue reservation.

## Author Contributions

MTL: writing original manuscript. EDN: supervision. VN:data analysis. LR: data collection. SAM: writing original manuscript. FS: data curation. MC: data collection. ML: data analysis. GL: supervision. NL: supervision.

## Conflict of Interest

The authors declare that the research was conducted in the absence of any commercial or financial relationships that could be construed as a potential conflict of interest.

## Publisher’s Note

All claims expressed in this article are solely those of the authors and do not necessarily represent those of their affiliated organizations, or those of the publisher, the editors and the reviewers. Any product that may be evaluated in this article, or claim that may be made by its manufacturer, is not guaranteed or endorsed by the publisher.
